# Primary Orbital Conjunctival Cyst Associated With the Inferior Rectus Muscle

**DOI:** 10.7759/cureus.71383

**Published:** 2024-10-13

**Authors:** Jonnah Kristina C Teope, Tatsuro Yokoyama, Yasuhiro Takahashi

**Affiliations:** 1 Oculoplastic, Orbital and Lacrimal Surgery, Aichi Medical University Hospital, Aichi, JPN; 2 Ophthalmology, TMG Asaka Medical Center, Saitama, JPN

**Keywords:** conjunctival cyst, diplopia, inferior rectus muscle, limited infraduction, primary

## Abstract

Primary simple conjunctival cysts are congenital, non-neoplastic, fluid-filled lesions lined by non-keratinizing squamous epithelium without dermal appendages. They are usually located in the anterior superonasal or superotemporal orbit and are typically managed with complete surgical excision. A 69-year-old woman presented with a 10-month history of diplopia and a 2-week history of right lower eyelid swelling. There was no history of any ocular trauma or surgery. Slit-lamp examination of the right eye revealed a greyish subconjunctival cyst located inferiorly. A Hess chart showed a limitation of infraduction in the right eye, and the field of binocular single vision presented diplopia in the lower and right-lower directions. Magnetic resonance imaging demonstrated a non-enhancing homogenous lesion above the inferior oblique muscle. The course of the inferior rectus muscle was unclear. Complete surgical excision was performed via the swinging eyelid approach. The tumor was firmly attached to the inferior rectus muscle intraoperatively. Pathological examinations of the excised tumor showed a unilocular cyst lined with non-keratinizing squamous epithelium, and it was devoid of goblet cells, dermal appendages, and muscle fibers. On day one post-surgery, full and equal extraocular movements were observed, with no complaints of diplopia. At the one-year follow-up, the Hess chart confirmed improvement of the limited infraduction, and no tumor recurrence was noted.

## Introduction

Conjunctival cysts are non-neoplastic fluid-filled lesions lined by non-keratinizing squamous epithelium that account for 3% of all orbital cysts [1-4]. They are commonly classified into primary (congenital) or secondary (acquired), and simple (without dermal appendages) or dermoid (with dermal appendages) [1,5]. They are frequently located in the anterior superonasal or superotemporal orbit [1].

Primary conjunctival cysts result from excessive invagination of the caruncular epithelium or fornix during embryonic development [6]. Secondary conjunctival cysts often develop by the detachment of conjunctival epithelium during surgery or trauma [6]. These cysts typically present as small to medium-sized, painless masses that were thought to have minimal structural and functional impact [1,6]. However, depending on their location and size, they may result in visual disturbances, reduced ocular motility, or cosmetic deformities [4-7]. Complete surgical excision is the primary treatment, with recurrence being the main postoperative concern.

Six cases of primary conjunctival cysts attaching to the superior rectus/levator complex, causing ptosis and diplopia, had been reported [4,5,7]. To the best of our knowledge, these are the only primary conjunctival cysts associated with the extraocular muscles. Other conjunctival cysts attaching to the extraocular muscles were secondary to trauma, strabismus surgery, enucleation, scleral buckling, inflammation, or infection [6,8-10].

We herein report an unusual case of an inferiorly located conjunctival cyst adherent to the inferior rectus (IR) muscle without preceding ocular trauma or surgery.

## Case presentation

A 69-year-old woman presented with a 10-month history of diplopia. The patient noticed right lower eyelid swelling two weeks before referral to our service. There was no history of any ocular trauma or surgery. Past medical history was unremarkable, except for medical treatment for diabetes mellitus. Antibody testing for rheumatoid arthritis and other pathological conditions was not performed.

**Figure 1:**
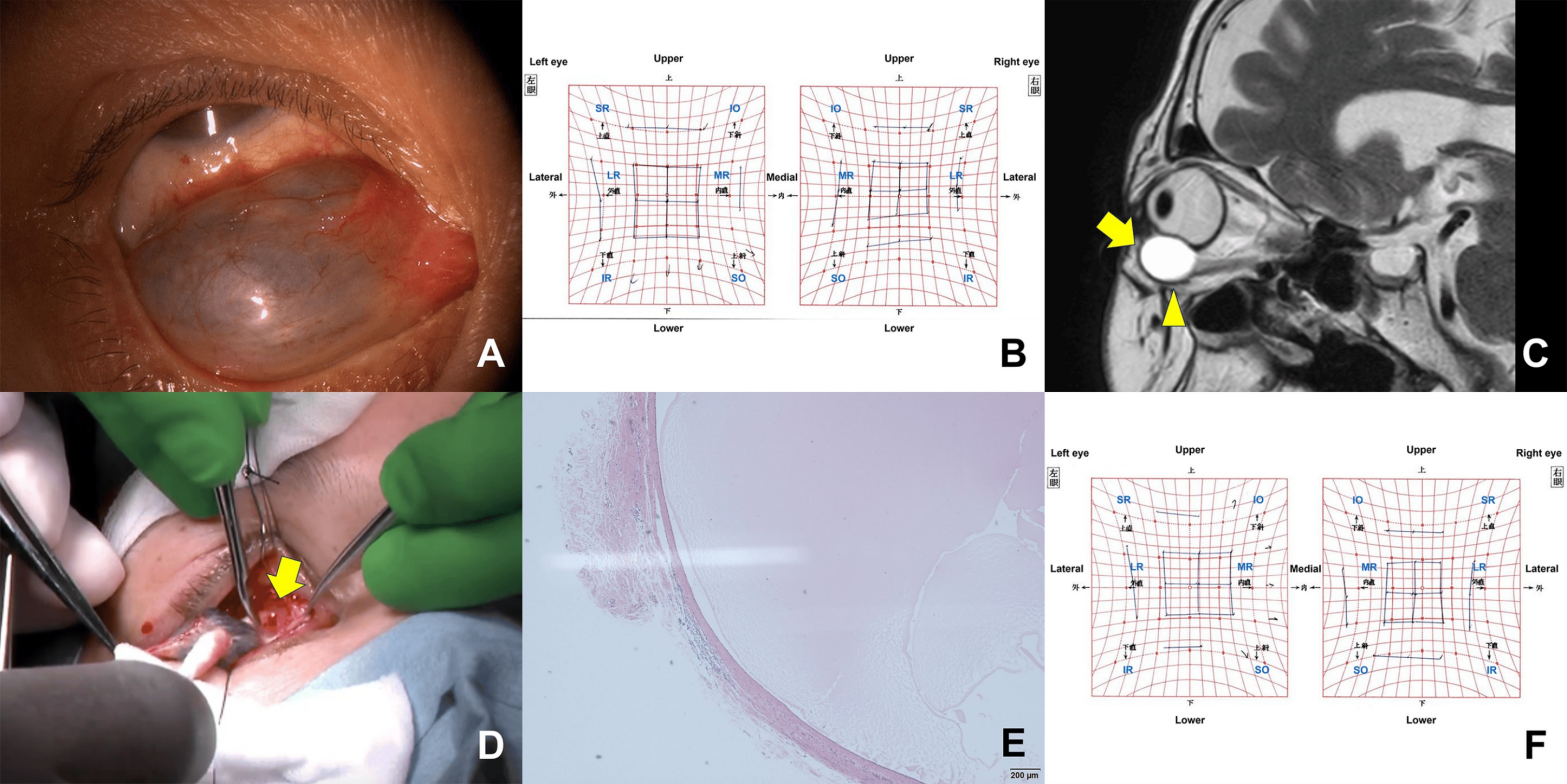
Photographs of the patient A. A slit-lamp photo showing a grayish subconjunctival cystic lesion. B. Preoperative Hess chart showing the limitation of infraduction in the right eye. English translation of the terms in Japanese on the Hess chart is added. C. A T2-weighted sagittal magnetic resonance image showing a homogenous high-intense mass (arrow) above the inferior oblique muscle (arrowhead). D. An intraoperative photo showing a mass attaching to the IR muscle (arrow). E. A pathological image showing a cyst lined with non-keratinizing squamous epithelium (hematoxylin & eosin; magnification, × 40). F. Postoperative Hess chart showing improvement of the limited infraduction. English translation of the terms in Japanese on the Hess chart is added. SR, superior rectus; IR, inferior rectus; MR, medial rectus; LR, lateral rectus; SO, superior oblique; IO, inferior oblique

On initial consultation, a slit-lamp examination of the right eye revealed a grayish subconjunctival cyst located inferiorly (Figure 1A). No other anterior or posterior segment abnormalities were observed. Decimal visual acuity was 1.0 in both eyes. Intraocular pressure was 11 mmHg in the right eye and 12 mmHg in the left eye. The Hess chart showed the limitation of infraduction in the right eye (Figure 1B), and the field of binocular single vision presented diplopia in the lower and right-lower directions. Hertel exophthalmometric value was 10.5 mm in the right eye and 12.5 mm in the left eye (base, 98 mm). T1- and T2-weighted magnetic resonance imaging (MRI) demonstrated a low- and high-intense homogenous lesion above the inferior oblique muscle, respectively (Figure 1C). The course of the IR muscle was unclear. Contrast-enhanced MRI showed no enhancement of the lesion.

Tumor excision was performed under local anesthesia via the swinging eyelid approach by two of the authors (TY and YT). The tumor was firmly attached to the IR muscle (Figure 1D). During the sharp dissection between the tumor and IR muscle, the muscle was partially lacerated. The muscle was sutured and approximated. Pathological examinations of the excised tumor showed a unilocular cyst lined with non-keratinizing squamous epithelium, and it was devoid of goblet cells, dermal appendages, and muscle fibers (Figure 1E).

Routine oral and topical antibiotics were given postoperatively. On day one post-surgery, full and equal extraocular movements were observed, with no complaints of diplopia. At the one-year follow-up, the Hess chart confirmed improvement of the limited infraduction (Figure 1F). The Hertel exophthalmometric value was 11 mm in the right eye and 13 mm in the left eye (base, 98 mm). Computed tomographic images revealed no recurrence of the tumor.

## Discussion

We present a rare case of a primary simple conjunctival cyst firmly adherent to the IR muscle. Conjunctival cysts are typically fixed to the tissue of origin [11]. The attachment of the cyst to the IR muscle, evidently seen intraoperatively, suggests that this is not an anteriorly located cyst causing a compressive effect. This is a lesion that expanded anteriorly from the IR muscle. Hence, the onset of diplopia occurred months prior to noticeable eyelid swelling and clinical hypoglobus, which may have contributed to the uneven Hertel exophthalmometric values.

The few reported cases of inferiorly located primary conjunctival cysts were superficial, with no attachment to surrounding tissues [3,12]. A conjunctival cyst adhering to the IR and inferior oblique muscles was noted after scleral buckling [8]. Similar cysts were noted to arise between operated extraocular muscles and sclera after strabismus surgery [6,9,10]. These secondary conjunctival cysts were postulated to arise from the deposition of conjunctival epithelial cells into deeper areas during suturing [8,9].

We classify our case as a primary conjunctival cyst. Primary conjunctival cysts are not attributed to trauma or surgery and may present from birth to the seventh decade of life, with symptoms lasting from months to years [3]. Considering the advanced age of the patient, we acknowledged the possibility of recall bias. However, we believe this is mitigated by the fact that major ocular traumas, like those described earlier, are unlikely to be forgotten.

Our case is comparable to the six reported cases of primary conjunctival cysts presenting with ptosis, diplopia, and eyelid swelling, although the order in which these symptoms appeared was not detailed [4,5,7]. These cysts were all found to be adherent to the superior rectus/levator complex [4,5,7]. Superior extraocular muscles arise from the superior mesodermal complex while the inferior muscles arise from the inferior mesodermal complex [13]. Sharing the same mesodermal origin and embryonic development, it is probable that the cyst presented herein also developed embryologically from the sequestration of the conjunctival epithelium into the orbit during the formation and progressive deepening of the fornices [3-7].

The MRI and histopathologic results of our case confirmed the diagnosis of a simple conjunctival cyst. MRI showed a non-enhancing cyst with no bone involvement while histopathologic examination revealed a unilocular cyst lined with non-keratinizing squamous epithelium without dermal appendages. These findings allow us to rule out both dermoid cysts and dermo-conjunctival cysts, which contain dermal appendages and typically show enhancing walls with bone erosion on imaging [1,14]. The absence of keratin further excludes the less common epidermo-conjunctival cyst, which contains both keratin and goblet cells [14]. While the presence of goblet cells supports the diagnosis of a conjunctival cyst, it is not definitive. Our case is similar to the reported cases of conjunctival cysts lacking goblet cells [15,16]. It is possible that a low count of goblet cells might have been missed during histopathological analysis. Nevertheless, the high T2 signal observed in this case suggests an aqueous component with mucus content, consistent with the role of goblet cells in mucus secretion.

Complete surgical excision remains the most preferred method of treatment for such cases. Cyst aspiration, thermal cautery, lasers, alcohol, or trichloroacetic acid injection had good outcomes for cysts with smaller diameters and no adhesion to surrounding tissues [15]. These modalities were associated with adjacent tissue damage and a higher rate of recurrence for larger and deeper lesions due to incomplete removal [17]. The swinging eyelid approach we performed allowed clear visualization of the entire cyst without the aid of any staining method, as some suggest [17]. Complete surgical excision without perforation or rupture was successfully done. However, due to its firm adherence to the IR muscle, the muscle was partly lacerated. No muscle fibers were noted in the post-excision pathology slide, affirming that no muscle was excised along with the cyst. In addition, meticulous suturing after excision is highly recommended to prevent secondary conjunctival cysts.

## Conclusions

To the best of our knowledge, this is the first case report of a primary simple conjunctival cyst associated with the IR muscle. This highlights the probability that conjunctival cysts may arise from other extraocular muscles without previous trauma or surgery. Eyelid swelling with motility restriction and/or diplopia should warrant suspicion of such cases. The diagnostic value of histopathologic examination and imaging is emphasized, as these cysts may be larger than visibly seen. Complete excision followed by meticulous suturing is essential to restore functionality and prevent recurrence.
